# Data mining of the essential causes of different types of fatal construction accidents

**DOI:** 10.1016/j.heliyon.2023.e13389

**Published:** 2023-02-02

**Authors:** Aminu Darda'u Rafindadi, Nasir Shafiq, Idris Othman, Ahmad Ibrahim, M.M. Aliyu, Miljan Mikić, Hamzh Alarifi

**Affiliations:** a Department of Civil & Environmental Engineering, Universiti Teknologi PETRONAS, 32610, Seri Iskandar, Perak Darul Ridzuan, Malaysia; b Department of Civil Engineering, Bayero University, Kano, P.M.B 3011, Kano State, Nigeria; c Department of Water Resources and Environmental Engineering, Ahmadu Bello University Zaria, Kaduna State, Nigeria; d Department of Engineering Management, University of Leeds, UK

**Keywords:** Data mining, Association rule, Fatal construction accidents, Types of construction accidents, Causes, Construction industry

## Abstract

Accident analysis is used to discover the causes of workplace injuries and devise methods for preventing them in the future. There has been little discussion in the previous studies of the specific elements contributing to deadly construction accidents. In contrast to previous studies, this study focuses on the causes of fatal construction accidents based on management factors, unsafe site conditions, and workers' unsafe actions. The association rule mining technique identifies the hidden patterns or knowledge between the root causes of fatal construction accidents, and one hundred meaningful association rules were extracted from the two hundred and fifty-three rules generated. It was discovered that many fatal construction accidents were caused by management factors, unsafe site circumstances, and risky worker behaviors. These analyses can be used to demonstrate plausible cause-and-effect correlations, assisting in building a safer working environment in the construction sector. The study findings can be used more efficiently to design effective inspection procedures and occupational safety initiatives. Finally, the proposed method should be tested in a broader range of construction situations and scenarios to ensure that it is as accurate as possible.

## 1 Introduction

Accident analysis is used to discover the causes of workplace injuries and devise methods for preventing them in the future [1]. It is thought that the only way to find any common or unifying factors in accident events is to look at aggregated accident data rather than just one case at a time [2]. Analysts have conventionally used statistical techniques to deduce patterns or information from acquired construction accident data. However, the methods are lengthy and sometimes biased. Construction is a data-heavy field that proliferates how much data it generates and how much data it collects [3]. Data mining methods are becoming more prevalent in the construction industry. Data mining techniques can get helpful information from a lot of data in the construction industry [4]. Data mining is a highly effective technique for discovering hidden knowledge from databases' vast amounts of complex data [5]. Patterns, correlations, linkages, and anomalies all fall under “hidden knowledge” [6]. Association rule mining is the widely used data mining approach in construction and other sectors. Association rules can be used to deduce the linkages and possible correlations between attributes in massive volumes of data [7]. Association rules are determined in the form of Q→R, where Q and R are disjoint item sets, Q is the antecedent, and R is the consequent. An association rule can help identify undiscovered relationships and produce an outcome that can be used for forecasting and decision-making [8].

The association rule mining technique identifies the hidden patterns or knowledge between the root causes of fatal construction accidents in Malaysia. In contrast to previous studies [9,10], this study focuses on specific causes of fatal construction accidents based on management factors, unsafe site conditions, and workers' unsafe actions in a country with different characteristics. There has been little discussion in the previous studies of the specific elements contributing to deadly construction accidents. Management factors, hazardous site conditions, and unsafe actions by workers are primary contributors to 302 fatal construction accidents in Malaysia. There is a dearth of research that adequately investigates the hidden patterns or knowledge of the root causes of fatal construction accidents in Malaysia. It is valid for different construction accidents identified from the historical accident data. Here is the structure of the rest of the paper, which follows. There is a review of the relevant literature in the next section. The methodology employed in the research is then presented herein. The study findings are then presented in the fourth section. Finally, conclusions of the leading research findings and practical implications are presented.

## 2 Association rule mining in the construction industry and other related fields

Construction accident rates continue to be a source of worry on a global scale. The construction sector has a higher rate of fatal accidents than other industries [11]. Construction accounts for one-third of all workplace deaths, and construction workers are six times likelier to die on the job than in other sectors [12]. The fatality rate for construction workers is three to six times higher than the national average [13]. Not only do construction accidents result in significant financial loss, but they frequently result in severe injury or death [4]. There is a paucity of studies into the root causes and contributing elements of construction site safety violations and accidents [14]. The first step in preventing accidents is to grasp the causal elements that influence their occurrence [15,16]. They tend to occur due to dissimilar factors and one or more management factors, unsafe actions, and conditions [10,[17], [18], [19], [20], [21], [22]].

Cheng et al. [10] used association rule mining to establish cause-effect correlations between occupational accident factors. They discovered that when a particular set of dangers occurs concurrently, a specific set of accidents is more likely to occur. Liao and Perng [9] also used association rule mining to determine occupational injury characteristics in the construction industry. Some patterns of occupational injuries in the construction industry have been discovered, and rain significantly impacts the number of fatalities. Association rule mining has been used to quantify the interdependence of construction flaws resulting in schedule delays, cost overruns, and quality degradation [23]. It was established that the proposed approach could systematically identify and quantify causal relationships between defect causes. Lee et al. [24] built a relational database of garment industry faults and extracted defect patterns using association rule mining. Verma et al. [25] used an association rule mining technique to deduce the cause-and-effect relationships underlying events in a steel mill. It could elicit frequent itemsets and association rules between observed unusual circumstances. Wang et al. [26] found patterns of defects in the transport industry by examining causal linkages between flaws in container cranes. Tong et al. [27] employed association rule mining to evaluate accident investigation records to determine the factors' interrelationships. Zeng et al. [7] also utilized association rule mining to examine mining cities' environmental, economic, and social features and their interrelationships.

The Apriori algorithm, the frequent pattern growth algorithm and the dynamic itemset counting algorithm are the most frequently utilized association rule mining algorithms [28]. Support, confidence, and lift are three critical indications of association rule mining [29]. Support is the percentage of the complete dataset covered by the rule, confidence measures the rule's inference's reliability, and lift measures the rule's antecedent and consequent interdependence. Effective strong regulations must meet three criteria [28]: a minimum level of support, a minimum level of confidence, and a minimum level of lift.

## 3 Methodology

Data mining techniques include association, correlation, classification, and clustering. This research mainly focuses on association rule mining using an apriori algorithm to determine the affinity between the management factors (MF), unsafe site conditions (USC), and workers' dangerous actions (WUA) of the seven identified types of construction accidents from the department of safety and health (DOSH) accident reports, Malaysia. Association rule mining is a data mining technique that allows the discovery of several rules or combinations of items or frequent itemsets. Association Rule Mining is defined as [30]: “*Let* $I=\left\{i_{1},i_{2},\ldots ,i_{n}\right\}$ *be a set of n binary attributes called factors. Let* $C=\left\{c_{1},c_{2},\ldots ,c_{m}\right\}$ *be set of the causes of accidents called the database. Each cause in C has a unique identification number and contains a subset of the items in I. A rule is defined as an implication of the form* Q→R *where* Q,R⊆I, *and* Q∩R=∅*. The set of items Q and R are called antecedent and consequent on the right-hand-side of the rule, respectively.*” An itemset is a collection of items. If an itemset contains *k* items, it is referred to as a *k*-itemset. A group of two or more factors is called an itemset. A frequent itemset appears regularly in the dataset. A set of factors is considered regular if it meets a certain minimal level of support and confidence.

Numerous scholars have included quantitative methodologies in the accident investigation process, of which association rule mining is one. The Apriori algorithm is used in this study because it does well with small datasets [31,32]. Small data were the fatal construction accidents sampled from all construction accidents within the specified study period. Any small datasets (e.g., 50) that can give a meaningful pattern are considered adequate to provide reliable results. It was developed by Agrawal [33] and Sriknat [34]. The apriori algorithm is the most often used for mining association rules [30]. The Apriori algorithm divides the rule mining process into two stages [35]. The database is scanned to identify all itemsets with support values greater than or equal to the predetermined minimum. Apriori uses an iterative technique called level-wise search, in which *k*-itemsets are utilized to explore (*k* + 1)-itemsets.

The set of common 1-itemsets is discovered by scanning the database for each item and collecting those that satisfy minimal support. *F*_*1*_ represents the resulting set. *F*_*1*_ is then used to locate *F*_*2*_, the set of frequent 2-itemsets, which is then used to locate *F*_*3*_, and so on, until no other frequent *k*-itemsets can be found. Each *F*_*k*_ requires a complete scan of the database. Second, a rule is made if it meets a certain confidence level. Rule support and confidence are two ways to measure how interesting a rule is. Support of rule *Q* → *R* is ‘s' if s% of the transactions in *T* contains the set *Q* ∪ *R*. Confidence of the rule *Q* → *R* is ‘c' if c% of the transactions in *T* that include the set *Q* also contains the set *R*. Strong rules are those that fulfill both minimum support and a minimum confidence criterion [30]. Many of the rules found through minimum support and confidence thresholds aren't attractive to the people who use them, even though they help eliminate many of the rules that aren't interesting [36]. A lift can be used to deal with this weakness. Lift is a simple way to measure how well two things are linked. It means that if the lift is less than 1, the occurrence of *Q* and *R* are mutually exclusive. However, if it is more than 1, the occurrence of *Q* indicates that the other occurs. There is no association between *Q* and *R* if the calculated lift equals 1 [35]. The second phase is easier to do, but the first step is more challenging [37]. Because the first step determines the overall performance of mining association rules, most studies focus on the first challenge. Apriori algorithm was used to determine the affinity between the 189 identified factors that causes the seven types of construction accidents, with 27 assigned to each. The procedure adopted in this research included five steps and is explained in the following sub-sections.

### 3.1 Data acquisition

Data were extracted from DOSH accident investigation reports, a critical literature evaluation, and experts' opinions to create an initial database. The fatal accidents in Malaysian construction from 2010 to 2019 were used as data sources. The research was approved by the Department of Civil and Environmental Engineering, Universiti Teknologi Petronas, informed consent was obtained, and the study complied with all regulations. This study considered a total of 302 fatal construction accidents. There is no benchmark for the minimum dataset for data mining, but experts argue that 50 and above fields could provide an excellent result [38]. Thus, the dataset used in this study is representative. The repercussions of deadly accidents are exhaustively and objectively detailed in accident investigation reports, which increases the reliability of the association rule mining results. An integrative review process was adopted to determine the relevant factors and sub-factors used in this study. The experts' opinions were gathered to assess the relevance of the sub-factors that contributed to the identified construction accident until they all agreed on the chosen sub-factors unanimously. They include a construction manager with ten years of expertise, an academic and project manager with more than fifteen years of experience, and a safety and health officer with more than a century of experience. Table 1, Table 2, Table 3 presented the lists of management factors, unsafe site conditions, and unsafe worker behaviours.

**Table 1:** List of management factors (MF).

Label	Management factors	Types of accident	Reference(s)	DOSH report	Expert(s)
**MF**_**1**_	Employment of unskilled personnel	All types	[[39], [40], [41], [42]]		✓
**MF**_**2**_	Not identifying, assessing, and controlling risks	All types	[39,43,44]	✓	✓
**MF**_**3**_	Failure to provide no-smoking signs at appropriate locations on-site	Fire and explosion	[45]	✓	✓
**MF**_**4**_	Not putting a safety data sheet (SDS) on the product's container with the hazard information	Chemical exposure	[46,47]	✓	✓
**MF**_**5**_	Financial constraints	All types	[[39], [40], [41]]		✓
**MF**_**6**_	Inadequate first aid measures	All types	[39]		✓
**MF**_**7**_	Insufficiency of Skilled Project Managers	All types	[39]		✓
**MF**_**8**_	Lack of management commitment or negligence on safety	All types	[10,[40], [41], [42],^48^]	✓	✓
**MF**_**9**_	Lack of or ill planning	All types	[40,41,48]		✓
**MF**_**10**_	Top management's safety ignorance	All types	[39,42]		✓
**MF**_**11**_	Lack of safety management manuals	All types	[39]	✓	✓
**MF**_**12**_	Lack of safety regulations and enforcement	All types	[[39], [40], [41],^43^,^44^,^49^,^50^]	✓	✓
**MF**_**13**_	Lack of safety training system	All types	[[39], [40], [41],^43^,^44^,^49^,^50^]	✓	✓
**MF**_**14**_	Lack of strict on-site safety supervision and poor management	All types	[10,51], [[39], [40], [41],^43^,^44^,^51^]	✓	✓
**MF**_**15**_	Lack of technical guidance in the related field	All types	[39]	✓	✓
**MF**_**16**_	Inadequate provision of safety equipment	All types	[40,41,43,44]	✓	✓
**MF**_**17**_	The workers' site supervisors or managers pushed them to speed up work on-site.	All types	[52]		✓
**MF**_**18**_	Deployment of faulty/unsafe tools, vehicles, and machines	All types	[40,41]	✓	✓
**MF**_**19**_	Management team behavior	All types	[40,41,43,44]		✓
**MF**_**20**_	Nonchalant attitude by the top management about safety	All types	[40,41,43,44]		✓
**MF**_**21**_	Lack of standard safety management system to guide supervisors on-site	All types	[[39], [40], [41], [42]]	✓	✓
**MF**_**22**_	Weak method for quality control	All types	[40,41,43,44]		✓
**MF**_**23**_	Overload and misaligned assignments	All types	[[39], [40], [41]]	–	✓
**MF**_**ex (FfH)**_	Deployment of substandard scaffolding or ladders	Fall from height	–	–	✓
**MF**_**ex (D/A)**_	Failure to provide an appropriate temporary framework for working in water	Drowning/asphyxiation	–	–	✓

**Table 2:** List unsafe site conditions (USC).

Label	Unsafe site conditions	Type of accident	Reference(s)	DOSH report	Expert(s)
**USC**_**1**_	Scaffolding/work platforms collapsing	Fall from height, struck-by, caught-in-between	[16,[53], [54], [55]]	✓	✓
**USC**_**2**_	Apparel hazards (e.g., protective clothing, hand cloves)	Caught-in-between, electrocution	[10,41,56,57]	✓	✓
**USC**_**3**_	Workplace congestedness	All types	[10,41,56,57]		✓
**USC**_**4**_	Faulty and unsafe electrical equipment or machinery	Fire and explosion	[58]	✓	✓
**USC**_**5**_	Faulty construction machines or trucks	Caught-in-between	[58]	✓	✓
**USC**_**6**_	Hoisting or lifting equipment or machine failure	Struck-by, caught-in-between	[16,53,59]	✓	✓
**USC**_**7**_	Faulty ladders and hoists	Fall from height	[10,48]	✓	✓
**USC**_**8**_	Poorly constructed makeshift waterwork platforms	Drowning/asphyxiation	[60]		✓
**USC**_**9**_	Defective tools/equipment/supplies/personal protective equipment	All types	[10,41,56,57]	✓	✓
**USC**_**10**_	Insufficient illumination during the night shifts	All types	[61]		✓
**USC**_**11**_	Incorrect labelled or unlabeled containers carrying hazardous chemicals	Chemical exposure	[62,63]	✓	✓
**USC**_**12**_	Signs for dangerous chemicals that are wrongly labelled or not labelled at all	Chemical exposure	[47,63]	✓	✓
**USC**_**13**_	Insufficient scaffolding	Fall from height	[10,44,48]	✓	✓
**USC**_**14**_	Insufficient warning systems	All types	[10,41,56,57]	✓	✓
**USC**_**15**_	Complex job on faulty scaffolding or ladders at height	Fall from height	[50,[64], [65], [66], [67], [68], [69], [70]]	✓	✓
**USC**_**16**_	Used hazardous chemicals not properly disposed	Chemical exposure	[62,63]	✓	✓
**USC**_**17**_	Lack of or improper on-site storage for flammable liquids or gases	Fire and explosion	[71]		✓
**USC**_**18**_	Insufficient or poor chemical storage	Chemical exposure	[71]	✓	✓
**USC**_**19**_	Lack of or improper on-site storage of explosives for blasting	Fire and explosion	[71]		✓
**USC**_**20**_	Lack of safety net and workers' tool belt while working at height	Struck-by	[17,71,72]	✓	✓
**USC**_**21**_	Lack of scaffolding toe boards	Struck-by	[17,71]	✓	✓
**USC**_**22**_	Loud and excessive noise	All types	[40,41,43,44]		✓
**USC**_**23**_	Poor housekeeping	All types	[73]	✓	✓
**USC**_**24**_	Poor storage and stalking	Struck-by	[17,71]		✓
**USC**_**25**_	Presence of combustible dust on-site	Fire and explosion	[60]		✓
**USC**_**26**_	Presence of electrical hazard on-site	Electrocution, Fire and explosion	[60]	✓	✓
**USC**_**27**_	Task difficulty or job complexity distracts personnel at heights.	Fall from height	[74]	✓	✓
**USC**_**28**_	Trench cave-ins during excavation	Caught-in-between	[16]	✓	✓
**USC**_**29**_	Unguarded edges/holes, slippery surfaces, skylights	Fall from height	[10,41,44,48,56,57], [43,75,76]	✓	✓
**USC**_**30**_	Unsafe building windows	Fall from height	[10,48]		✓
**USC**_**31**_	Unsafe environmental conditions	All types	[73]		✓
**USC**_**32**_	Procedures for work and operation that are unsafe	All types	[40,41,77]	✓	✓
**USC**_**ex (CiBA)**_	Too many blind spots on-site	Caught-in-between accidents	–	–	✓
**USC**_**ex1(D/A)**_	Accidental slab or cover collapse in a tight space	Drowning/asphyxiation	–	–	✓
**USC**_**ex2(D/A)**_	High-tide flow	Drowning/asphyxiation	–	–	✓
**USC**_**ex3(D/A)**_	Job difficulty involving water or enclosed area	Drowning/asphyxiation	–	–	✓
**USC**_**ex4(D/A)**_	Existence of harmful gases in a confined area	Drowning/asphyxiation	–	–	✓
**USC**_**ex5(D/A)**_	Weather conditions while operating in bodies of water	Drowning/asphyxiation	–	–	✓
**USC**_**ex1 (ELEC)**_	Defective existing wiring	Electrocution	–	–	✓
**USC**_**ex2(ELEC)**_	Loading and unloading construction materials close to live electric wire	Electrocution	–	–	✓

**Table 3:** List of workers' unsafe actions (WUA).

Label	Workers' unsafe actions	Type of accident	Reference(s)	DOSH report	Expert(s)
**WUA**_**1**_	Boisterous play among the workers	All types	[57]		✓
**WUA**_**2**_	Carelessness/negligence	All types	[10,[39], [40], [41],^51^,^57^,^71^,[78], [79], [80]]	✓	✓
**WUA**_**3**_	Non-compliance with hoisting or lifting procedures	Struck-by, caught-in-between	[40,57,71]	✓	✓
**WUA**_**4**_	Contravention of norms for safe working procedures	All types	[10,[39], [40], [41],^51^,^57^,^71^,[78], [79], [80]]	✓	✓
**WUA**_**5**_	PPE not worn or used in the wrong way	All types	[10,40,41,43,44,48,50,51,81]	✓	✓
**WUA**_**6**_	Repairing machinery or equipment in motion	Caught-in-between	[57]	✓	✓
**WUA**_**7**_	Mistakes made by workers and the wrong use of controls	All types	[49]	✓	✓
**WUA**_**8**_	Improper handling of explosives for blasting	Fire and explosion	[82]		✓
**WUA**_**9**_	Improper handling of hazardous chemicals	Chemical exposure	[62,63]	✓	✓
**WUA**_**10**_	Personal qualities (such as a worker's safety attitude)	All types	[43,44,57,71,75,83]	✓	✓
**WUA**_**11**_	Insufficient safety understanding and misjudging dangerous scenarios	All types	[10,[39], [40], [41], [42],^48^,^49^,^51^,^57^,^71^,^78^,^79^,[84], [85], [86], [87]]	✓	✓
**WUA**_**12**_	Operating equipment or machines without qualification or authorization	Struck-by, caught-in-between	[40,57]	✓	✓
**WUA**_**13**_	Operating machines at an unacceptable speed	Caught-in-between	[57]	✓	✓
**WUA**_**14**_	Physical and emotional stress	All types	[84]	✓	✓
**WUA**_**15**_	Rushing to complete the work	All types	[51]	✓	✓
**WUA**_**16**_	Using dangerous methods or steps	Chemical exposure	[62,63]	✓	✓
**WUA**_**17**_	Dangerous behaviour of a third party(s)	All types	[57]	✓	✓
**WUA**_**18**_	Use of faulty or unsafe electrical equipment or machinery	Electrocution, Fire and explosion	[40,41]	✓	✓
**WUA**_**19**_	Incorrect or inadequate personal protective equipment use	All types	[49]	✓	✓
**WUA**_**20**_	Utilization of risky methods or procedures	All types	[40,41,43,44]	✓	✓
**WUA**_**21**_	Voluntarily doing risky activities	All types	[75]	✓	✓
**WUA**_**22**_	Work in a hazardous position or posture	All types	[57]	✓	
**WUA**_**ex(CE)**_	Unauthorized access to hazardous chemicals	Chemical exposure	–	–	✓
**WUA**_**ex(F&E)**_	Unauthorized access to explosives for blasting	Fire and explosion	–	–	✓

### 3.2 Data preparation

It is the process of transforming raw data into a format that can be analyzed efficiently and adequately.

The dataset contains seven rows and twenty-seven columns (see Table 4). The identified and utilized management factors, unsafe site conditions, and workers' unsafe actions that caused various fatal construction accidents for the algorithm development were based on the DOSH report and experts' opinions. Fall from height (FfH) is the most common fatal construction accident with about 37%, followed by struck-by accident (SbA) with about 31%, and caught-in-between accident (CiBA) with 18%. Others include drowning/asphyxiation (D/A) with about 10%, electrocution (ELEC) with about 3%, chemical exposure (CE) with about 1%, and fire and explosion (F&E) with less than 1%.

**Table 4:** Dataset for Apriori Algorithm (AA) development.

Code	Management factors (MF)									Unsafe site conditions (USC)									Workers' unsafe actions (WUA)								
**FfH**	MF_13_	MF_5_	MF_1_	MF_16_	MF_21_	MF_ex_	MF_14_	MF_12_	MF_2_	USC_3_	USC_23_	USC_15_	USC_32_	USC_29_	USC_9_	USC_1_	USC_7_	USC_13_	WUA_15_	WUA_2_	WUA_10_	WUA_5_	WUA_11_	WUA_20_	WUA_1_	WUA_4_	WUA_19_
**SbA**	MF_13_	MF_5_	MF_1_	MF_16_	MF_2_	MF_18_	MF_12_	MF_14_	MF_21_	USC_3_	USC_23_	USC_1_	USC_24_	USC_6_	USC_9_	USC_32_	USC_20_	USC_21_	WUA_10_	WUA_2_	WUA_14_	WUA_5_	WUA_11_	WUA_20_	WUA_12_	WUA_3_	WUA_19_
**CiBA**	MF_13_	MF_5_	MF_1_	MF_16_	MF_2_	MF_18_	MF_12_	MF_14_	MF_21_	USC_3_	USC_23_	USC_32_	USC_5_	USC_6_	USC_9_	USC_1_	USC_28_	USC_ex_	WUA_10_	WUA_2_	WUA_13_	WUA_6_	WUA_11_	WUA_20_	WUA_12_	WUA_3_	WUA_19_
**D/A**	MF_13_	MF_5_	MF_1_	MF_16_	MF_21_	MF_ex_	MF_14_	MF_12_	MF_2_	USC_ex1_	USC_32_	USC_ex2_	USC_ex3_	USC_8_	USC_9_	USC_ex4_	USC_ex5_	USC_10_	WUA_21_	WUA_2_	WUA_10_	WUA_5_	WUA_11_	WUA_20_	WUA_4_	WUA_14_	WUA_19_
**ELEC**	MF_13_	MF_5_	MF_1_	MF_16_	MF_21_	MF_15_	MF_14_	MF_12_	MF_2_	USC_3_	USC_23_	USC_32_	USC_ex1_	USC_26_	USC_9_	USC_ex2_	USC_14_	USC_2_	WUA_10_	WUA_2_	WUA_15_	WUA_5_	WUA_11_	WUA_20_	WUA_4_	WUA_7_	WUA_19_
**CE**	MF_13_	MF_5_	MF_1_	MF_16_	MF_21_	MF_4_	MF_14_	MF_12_	MF_2_	USC_3_	USC_23_	USC_32_	USC_16_	USC_31_	USC_9_	USC_11_	USC_12_	USC_18_	WUA_10_	WUA_2_	WUA_15_	WUA_5_	WUA_11_	WUA_20_	WUA_ex_	WUA_9_	WUA_19_
**F&E**	MF_13_	MF_5_	MF_1_	MF_16_	MF_21_	MF_3_	MF_14_	MF_12_	MF_2_	USC_3_	USC_23_	USC_32_	USC_25_	USC_26_	USC_9_	USC_4_	USC_17_	USC_19_	WUA_10_	WUA_2_	WUA_14_	WUA_5_	WUA_11_	WUA_20_	WUA_ex_	WUA_8_	WUA_19_

**Key –** FfH = Fall from height, SbA = Struck-by accident, CiBA = Caught-in-between accident, D/A = Drowning/Asphyxiation, ELEC = Electrocution, CE = Chemical exposure, F&E = Fire and explosion.

### 3.3 Support and confidence threshold setting

Determining support and confidence levels is critical in the association rule mining procedure. When thresholds are set too low, irrelevant rules are generated, whereas no rules are developed when they are set too high [27]. Support threshold filters out the sub-factors that are not frequent in the dataset and is calculated using the formula in equation (1). The minimum support in this research is 2 (28.57%). The criteria for choosing the minimum support of 28.57% for this study is because a minimum of two factors can cause fatal construction accidents based on the abstract from the DOSH report. Confidence is used to confirm the percentage of cases in which the rules generated are valid. Based on the accident report, the likelihood of two factors or more causing fatal construction accidents is greater than 50%. The minimum confidence is set at 60% and is computed using the formula provided in equation (2).

(1) $$Support\left(Q\right)=\frac{Number\;of\;accidents\;in\;which\;factor\;Q\;appears}{Total\;number\;of\;accident\;occurred}$$

(2) $$Confidence\left(Q\to R\right)=\frac{Support\left(QUR\right)}{Support\left(Q\right)}$$

### 3.4 Frequent items selection

Based on the set minimum support of 2 in this study, only the sub-factors whose count starts from two and above would be considered. The count begins with two until the maximum support of 7 to determine the most frequent sub-factors in the dataset.

### 3.5 Rules generation

The algorithm extracts rules from the frequent itemsets once all of them have been detected. The most common item sets are then translated into association rules representing how often the antecedent and consequent occur together. Association rules are written in the format: sub−factorQ→sub−factorR. This means you obtain a rule that tells you that if sub-factor Q causes an accident, then sub-factor R is also likely to cause the same thing.

### 3.6 Lift computation

The last step is to calculate the lift of each rule once all the rules are generated. Lift is a performance statistic for rules that indicates the strength of the relationship between the sub-factors. This means that lift compares an association rule's improvement to the total dataset. It is calculated using the formula given in equation (3). If a rule's lift is 1, the sub-factors are independent. If a rule's lift value is more than 1, it indicates how heavily the left-hand side sub-factor depends on the right-hand side. Any rule with a lift of one may be deleted. Meaningful rules must adhere to the lift >1 standard. But must fulfill the minimum support and confidence threshold.

(3) $$Lift=\frac{P\left(Q\cap R\right)}{P\left(Q\right)*P\left(R\right)}$$

## 4 Results and discussion

### 4.1 Results

The study scanned the dataset to identify the count of each sub-factor. Because of the limited number of rows for the dataset, we just skipped to the most frequent n – sub-factor sets, 22. The frequent 22 – sub-factor sets appear twice in the dataset, which is the minimum support for the apriori algorithm development (refer to equation (4)). And nonempty subsets are given in equation (5). Two hundred and fifty-three (253) rules were generated from the 22 – sub-factor sets, and a hundred (100) demonstrate strong relationships among the sub-factors based on the lift of greater than 1. The study considers superficial and deep factors that may contribute to the emergence of scenarios or situations that cause accidents. One of the goals is to increase awareness of the accident causation process, aid in conducting organized accident investigations, and provide advice on effective accident prevention methods (e.g., safety training and job hazard identification). No attempt has been made to identify the association rules between the sub-factors that cause different types of construction accidents. There is still a dearth of detail in accident causation models regarding the factors contributing significantly to construction accidents. Effective mitigation of causal factors involves a greater understanding of the most significant factors, who can reasonably be expected to manage them, and how such control may be accomplished most successfully.

(4) $$\{MF2,MF_{5},MF_{12},MF_{13},MF_{14},MF_{16},MF_{18},MF2_{1},USC_{1}\text{,}USC3,USC6,USC9,USC23,USC32,WUA2,WUA3,WUA10,WUA11,WUA12,WUA19,WUA20\}$$

Nonempty subsets are:

{USC9},{USC23},{USC32},{WUA2},{WUA3},{WUA10},{WUA11},{WUA12},{WUA19},{WUA20},

$$\left\{MF_{1,}MF2\right\},\left\{MF_{1,}MF_{5}\right\},\left\{MF_{1,}MF_{12}\right\},\left\{MF_{1,}MF_{13}\right\},\left\{MF_{1,}MF_{14}\right\},\left\{MF_{1,}MF_{16}\right\},\left\{MF_{1,}MF_{18}\right\},\left\{MF_{1,}MF2_{1}\right\}\text{,}$$

$$\left\{MF_{1,}USC_{1}\right\},\left\{MF_{1,}USC3\right\},\left\{MF_{1,}USC6\right\},\left\{MF_{1,}USC9\right\},\left\{MF_{1,}USC23\right\},\left\{MF_{1,}USC32\right\},\left\{MF_{1,}WUA2\right\}\text{,}$$

$$\left\{MF_{1,}WUA3\right\},\left\{MF_{1,}WUA10\right\},\left\{MF_{1,}WUA11\right\},\left\{MF_{1,}WUA12\right\},\left\{MF_{1,}WUA19\right\},\left\{MF_{1,}WUA20\right\}$$

$$\left\{MF2,MF_{5}\right\},\left\{MF2,MF_{12}\right\},\left\{MF2,MF_{13}\right\},\left\{MF2,MF_{14}\right\},\left\{MF2,MF_{16}\right\},\left\{MF2,MF_{18}\right\},\left\{MF2,MF2_{1}\right\},\left\{MF2,USC_{1}\right\}\text{,}$$

{MF2,USC3},{MF2,USC6},{MF2,USC9},{MF2,USC23},{MF2,USC32},{MF2,WUA2},{MF2,WUA3},

{MF2,WUA10},{MF2,WUA11},{MF2,WUA12},{MF2,WUA19},{MF2,WUA20},

$$\left\{MF_{5,}MF_{12}\right\},\left\{MF_{5,}MF_{13}\right\},\left\{MF_{5,}MF_{14}\right\},\left\{MF_{5,}MF_{16}\right\},\left\{MF_{5,}MF_{18}\right\},\left\{MF_{5,}MF2_{1}\right\},\left\{MF_{5,}USC_{1}\right\}\text{,}$$

$$\left\{MF_{5,}USC3\right\},\left\{MF_{5,}USC6\right\},\left\{MF_{5,}USC9\right\},\left\{MF_{5,}USC23\right\},\left\{MF_{5,}USC32\right\},\left\{MF_{5,}WUA2\right\},\left\{MF_{5,}WUA3\right\}\text{,}$$

$$\left\{MF_{5,}WUA10\right\},\left\{MF_{5,}WUA11\right\},\left\{MF_{5,}WUA12\right\},\left\{MF_{5,}WUA19\right\},\left\{MF_{5,}WUA20\right\}\text{,}$$

$$\left\{MF_{12,}MF_{13}\right\},\left\{MF_{12,}MF_{14}\right\},\left\{MF_{12,}MF_{16}\right\},\left\{MF_{12,}MF_{18}\right\},\left\{MF_{12,}MF2_{1}\right\},\left\{MF_{12,}USC_{1}\right\},\left\{MF_{12,}USC3\right\}\text{,}$$

$$\left\{MF_{12,}USC6\right\},\left\{MF_{12,}USC9\right\},\left\{MF_{12,}USC23\right\},\left\{MF_{12,}USC32\right\},\left\{MF_{12,}WUA2\right\},\left\{MF_{12,}WUA3\right\}\text{,}$$

$$\left\{MF_{12,}WUA10\right\},\left\{MF_{12,}WUA11\right\},\left\{MF_{12,}WUA12\right\},\left\{MF_{12,}WUA19\right\},\left\{MF_{12,}WUA20\right\}\text{,}$$

$$\left\{MF_{13,}MF_{14}\right\},\left\{MF_{13,}MF_{16}\right\},\left\{MF_{13,}MF_{18}\right\},\left\{MF_{13,}MF2_{1}\right\},\left\{MF_{13,}USC_{1}\right\},\left\{MF_{13,}USC3\right\},\left\{MF_{13,}USC6\right\}\text{,}$$

$$\left\{MF_{13,}USC9\right\},\left\{MF_{13,}USC23\right\},\left\{MF_{13,}USC32\right\},\left\{MF_{13,}WUA2\right\},\left\{MF_{13,}WUA3\right\},\left\{MF_{13,}WUA10\right\}\text{,}$$

$$\left\{MF_{13,}WUA11\right\},\left\{MF_{13,}WUA12\right\},\left\{MF_{13,}WUA19\right\},\left\{MF_{13,}WUA20\right\}\text{,}$$

$$\left\{MF_{14,}MF_{16}\right\},\left\{MF_{14,}MF_{18}\right\},\left\{MF_{14,}MF2_{1}\right\},\left\{MF_{14,}USC_{1}\right\},\left\{MF_{14,}USC3\right\},\left\{MF_{14,}USC6\right\},\left\{MF_{14,}USC9\right\}\text{,}$$

$$\left\{MF_{14,}USC23\right\},\left\{MF_{14,}USC32\right\},\left\{MF_{14,}WUA2\right\},\left\{MF_{14,}WUA3\right\},\left\{MF_{14,}WUA10\right\},\left\{MF_{14,}WUA11\right\}\text{,}$$

$$\left\{MF_{14,}WUA12\right\},\left\{MF_{14,}WUA19\right\},\left\{MF_{14,}WUA20\right\}\text{,}$$

$$\left\{MF_{16,}MF_{18}\right\},\left\{MF_{16,}MF2_{1}\right\},\left\{MF_{16,}USC_{1}\right\},\left\{MF_{16,}USC3\right\},\left\{MF_{16,}USC6\right\},\left\{MF_{16,}USC9\right\},\left\{MF_{16,}USC23\right\}\text{,}$$

$$\left\{MF_{16,}USC32\right\},\left\{MF_{16,}WUA2\right\},\left\{MF_{16,}WUA3\right\},\left\{MF_{16,}WUA10\right\},\left\{MF_{16,}WUA11\right\},\left\{MF_{16,}WUA12\right\}\text{,}$$

$$\left\{MF_{16,}WUA19\right\},\left\{MF_{16,}WUA20\right\}\text{,}$$

$$\left\{MF_{18,}MF2_{1}\right\},\left\{MF_{18,}USC_{1}\right\},\left\{MF_{18,}USC3\right\},\left\{MF_{18,}USC6\right\},\left\{MF_{18,}USC9\right\},\left\{MF_{18,}USC23\right\},\left\{MF_{18,}USC32\right\}\text{,}$$

$$\left\{MF_{18,}WUA2\right\},\left\{MF_{18,}WUA3\right\},\left\{MF_{18,}WUA10\right\},\left\{MF_{18,}WUA11\right\},\left\{MF_{18,}WUA12\right\}\text{,}$$

$$\left\{MF_{18,}WUA19\right\},\left\{MF_{18,}WUA20\right\}\text{,}$$

$$\left\{MF2_{1,}USC_{1}\right\},\left\{MF2_{1,}USC3\right\},\left\{MF2_{1,}USC6\right\},\left\{MF2_{1,}USC9\right\},\left\{MF2_{1,}USC23\right\},\left\{MF2_{1,}USC32\right\},\left\{MF2_{1,}WUA2\right\}\text{,}$$

$$\left\{MF2_{1,}WUA3\right\},\left\{MF2_{1,}WUA10\right\},\left\{MF2_{1,}WUA11\right\},\left\{MF2_{1,}WUA12\right\},\left\{MF2_{1,}WUA19\right\},\left\{MF2_{1,}WUA20\right\}\text{,}$$

$$\left\{USC_{1,}USC3\right\},\left\{USC_{1,}USC6\right\},\left\{USC_{1,}USC9\right\},\left\{USC_{1,}USC23\right\},\left\{USC_{1,}USC32\right\},\left\{USC_{1,}WUA2\right\},\left\{USC_{1,}WUA3\right\}\text{,}$$

$$\left\{USC_{1,}WUA10\right\},\left\{USC_{1,}WUA11\right\},\left\{USC_{1,}WUA12\right\},\left\{USC_{1,}WUA19\right\},\left\{USC_{1,}WUA20\right\}\text{,}$$

{USC3,USC6},{USC3,USC9},{USC3,USC23},{USC3,USC32},{USC3,WUA2},{USC3,WUA3},{USC3,WUA10},

{USC3,WUA11},{USC3,WUA12},{USC3,WUA19},{USC3,WUA20},

{USC6,USC9},{USC6,USC23},{USC6,USC32},{USC6,WUA2},{USC6,WUA3},{USC6,WUA10},{USC6,WUA11},

{USC6,WUA12},{USC6,WUA19},{USC6,WUA20},

{USC9,USC23},{USC9,USC32},{USC9,WUA2},{USC9,WUA3},{USC9,WUA10},{USC9,WUA11},

{USC9,WUA12},{USC9,WUA19},{USC9,WUA20},

{USC23,USC32},{USC23,WUA2},{USC23,WUA3},{USC23,WUA10},{USC23,WUA11},{USC23,WUA12},

{USC23,WUA19},{USC23,WUA20},

{USC32,WUA2},{USC32,WUA3},{USC32,WUA10},{USC32,WUA11},{USC32,WUA12},{USC32,WUA19},

{USC32,WUA20},{WUA2,WUA3},{WUA2,WUA10},{WUA2,WUA11},{WUA2,WUA12},

{WUA2,WUA19},{WUA2,WUA20},

{WUA3,WUA10},{WUA3,WUA11},{WUA3,WUA12},{WUA3,WUA19},{WUA3,WUA20},

{WUA10,WUA11},{WUA10,WUA12},{WUA10,WUA19},{WUA10,WUA20},

{WUA11,WUA12},{WUA11,WUA19},{WUA11,WUA20},

{WUA12,WUA19},{WUA12,WUA20},

(5) {WUA19,WUA20}}

The study findings indicate patterns in the causes of fatal accidents on construction sites. The hundred meaningful rules that were generated are explained as follows. Five one-item rules and ninety-five two-item rules satisfy the minimum support of 28.57%, minimum confidence of 60%, and a lift of more than 1. The occurrence of the antecedent (sub-factor(s)) indicates the likely occurrence of other sub-factors (consequences) from the sub-set that could cause fatal construction accidents (refer to Table 5). For example, rule 8 indicates that 100% of fatal construction accidents may be attributable to management deploying faulty/unsafe tools, vehicles, and machines (MF_18_). Deploying defective/unsafe tools, vehicles, and machines (MF_18_) could lead to hazardous site conditions and workers' dangerous actions, usually because of limited resources for all seven construction accidents. Most of the management factors depend on the company's finances. Rule 10 indicates that 66.67% of fatal construction accidents are likely to occur because of scaffolding/working platforms (USC_1_). Rule 12 means that 100% of fatal construction accidents result from faulty hoisting or lifting equipment or machine (USC_6_). A collapse in scaffolding/working platforms (USC_1_) and faulty hoisting or lifting equipment or machine (USC_6_) could be due to other unsafe site conditions and workers' unsafe actions, mostly primarily if the faulty ones are provided by the management and construction workers lack the training to erect or dismantle them correctly. Rules 17 and 20 indicate that 100% of fatal construction accidents are due to non-compliance with hoisting or lifting procedures (WUA_3_) and operating equipment or machines without qualification or authorization (WUA_12_).

**Table 5:** Association rules with specified antecedent.

Rule ID	Antecedent	Consequence	Support (%)	Confidence (%)	Lift	Remark
**8**	{MF18}	→{N−MF18}	28.57%	100%	3.5	Valid
**10**	{USC1}	→{N−USC1}	28.57%	66.67%	2.3	Valid
**12**	{USC6}	→{N−USC6}	28.57%	100%	3.5	Valid
**17**	{WUA3}	→{N−WUA3}	28.57%	100%	3.5	Valid
**20**	{WUA12}	→{N−WUA12}	28.57%	100%	3.5	Valid
**29**	$\left\{MF_{1,}MF_{18}\right\}$	$\to \left\{N-\left\{MF_{1,}MF_{18}\right\}\right\}$	28.57%	100%	3.5	Valid
**31**	$\left\{MF_{1,}USC_{1}\right\}$	$\to \left\{N-\left\{MF_{1,}USC_{1}\right\}\right\}$	28.57%	66.67%	2.3	Valid
**33**	$\left\{MF_{1,}USC6\right\}$	$\to \left\{N-\left\{MF_{1,}USC6\right\}\right\}$	28.57%	100%	3.5	Valid
**38**	$\left\{MF_{1,}WUA3\right\}$	$\to \left\{N-\left\{MF_{1,}WUA3\right\}\right\}$	28.57%	100%	3.5	Valid
**41**	$\left\{MF_{1,}WUA12\right\}$	$\to \left\{N-\left\{MF_{1,}WUA12\right\}\right\}$	28.57%	100%	3.5	Valid
**49**	$\left\{MF2,MF_{18}\right\}$	$\to \left\{N-\left\{MF2,MF_{18}\right\}\right\}$	28.57%	100%	3.5	Valid
**51**	$\left\{MF2,USC_{1}\right\}$	$\to \left\{N-\left\{MF2,USC_{1}\right\}\right\}$	28.57%	66.67%	2.3	Valid
**53**	$\left\{MF2,USC_{6}\right\}$	$\to \left\{N-\left\{MF2,USC_{6}\right\}\right\}$	28.57%	100%	3.5	Valid
**58**	{MF2,WUA3}	→{N−{MF2,WUA3}}	28.57%	100%	3.5	Valid
**61**	{MF2,WUA12}	→{N−{MF2,WUA12}}	28.57%	100%	3.5	Valid
**68**	$\left\{MF_{5,}MF_{18}\right\}$	$\to \left\{N-\left\{MF_{5,}MF_{18}\right\}\right\}$	28.57%	100%	3.5	Valid
**70**	$\left\{MF_{5,}USC_{1}\right\}$	$\to \left\{N-\left\{MF_{5,}USC_{1}\right\}\right\}$	28.57%	66.67%	2.3	Valid
**72**	$\left\{MF_{5,}USC_{6}\right\}$	$\to \left\{N-\left\{MF_{5,}USC_{6}\right\}\right\}$	28.57%	100%	3.5	Valid
**77**	$\left\{MF_{5,}WUA3\right\}$	$\to \left\{N-\left\{MF_{5,}WUA3\right\}\right\}$	28.57%	100%	3.5	Valid
**80**	$\left\{MF_{5,}WUA12\right\}$	$\to \left\{N-\left\{MF_{5,}WUA12\right\}\right\}$	28.57%	100%	3.5	Valid
**86**	$\left\{MF_{12,}MF_{18}\right\}$	$\to \left\{N-\left\{MF_{12,}MF_{18}\right\}\right\}$	28.57%	100%	3.5	Valid
**88**	$\left\{MF_{12,}USC_{1}\right\}$	$\to \left\{N-\left\{MF_{12,}USC_{1}\right\}\right\}$	28.57%	66.67%	2.3	Valid
**90**	$\left\{MF_{12,}USC_{6}\right\}$	$\to \left\{N-\left\{MF_{12,}USC_{6}\right\}\right\}$	28.57%	100%	3.5	Valid
**94**	$\left\{MF_{12,}WUA2\right\}$	$\to \left\{N-\left\{MF_{12,}WUA2\right\}\right\}$	28.57%	100%	3.5	Valid
**98**	$\left\{MF_{12,}WUA12\right\}$	$\to \left\{N-\left\{MF_{12,}WUA12\right\}\right\}$	28.57%	100%	3.5	Valid
**103**	$\left\{MF_{13,}MF_{18}\right\}$	$\to \left\{N-\left\{MF_{13,}MF_{18}\right\}\right\}$	28.57%	100%	3.5	Valid
**105**	$\left\{MF_{13,}USC_{1}\right\}$	$\to \left\{N-\left\{MF_{13,}USC_{1}\right\}\right\}$	28.57%	66.67%	2.3	Valid
**107**	$\left\{MF_{13,}USC_{6}\right\}$	$\to \left\{N-\left\{MF_{13,}USC_{6}\right\}\right\}$	28.57%	100%	3.5	Valid
**111**	$\left\{MF_{13,}WUA2\right\}$	$\to \left\{N-\left\{MF_{13,}WUA2\right\}\right\}$	28.57%	100%	3.5	Valid
**115**	$\left\{MF_{13,}WUA12\right\}$	$\to \left\{N-\left\{MF_{13,}WUA12\right\}\right\}$	28.57%	100%	3.5	Valid
**119**	$\left\{MF_{14,}MF_{18}\right\}$	$\to \left\{N-\left\{MF_{14,}MF_{18}\right\}\right\}$	28.57%	100%	3.5	Valid
**121**	$\left\{MF_{14,}USC_{1}\right\}$	$\to \left\{N-\left\{MF_{14,}USC_{1}\right\}\right\}$	28.57%	66.67%	2.3	Valid
**123**	$\left\{MF_{14,}USC_{6}\right\}$	$\to \left\{N-\left\{MF_{14,}USC_{6}\right\}\right\}$	28.57%	100%	3.5	Valid
**128**	$\left\{MF_{14,}WUA3\right\}$	$\to \left\{N-\left\{MF_{14,}WUA3\right\}\right\}$	28.57%	100%	3.5	Valid
**131**	$\left\{MF_{14,}WUA12\right\}$	$\to \left\{N-\left\{MF_{14,}WUA12\right\}\right\}$	28.57%	100%	3.5	Valid
**134**	$\left\{MF_{16,}MF_{18}\right\}$	$\to \left\{N-\left\{MF_{16,}MF_{18}\right\}\right\}$	28.57%	100%	3.5	Valid
**136**	$\left\{MF_{16,}USC_{1}\right\}$	$\to \left\{N-\left\{MF_{16,}USC_{1}\right\}\right\}$	28.57%	66.67%	2.3	Valid
**138**	$\left\{MF_{16,}USC_{6}\right\}$	$\to \left\{N-\left\{MF_{16,}USC_{6}\right\}\right\}$	28.57%	100%	3.5	Valid
**143**	$\left\{MF_{16,}WUA3\right\}$	$\to \left\{N-\left\{MF_{16,}WUA3\right\}\right\}$	28.57%	100%	3.5	Valid
**146**	$\left\{MF_{16,}WUA12\right\}$	$\to \left\{N-\left\{MF_{16,}WUA12\right\}\right\}$	28.57%	100%	3.5	Valid
**149**	$\left\{MF_{18,}MF2_{1}\right\}$	$\to \left\{N-\left\{MF_{18,}MF2_{1}\right\}\right\}$	28.57%	100%	3.5	Valid
**150**	$\left\{MF_{18,}USC_{1}\right\}$	$\to \left\{N-\left\{MF_{18,}USC_{1}\right\}\right\}$	28.57%	100%	3.5	Valid
**151**	$\left\{MF_{18,}USC_{3}\right\}$	$\to \left\{N-\left\{MF_{18,}USC_{3}\right\}\right\}$	28.57%	100%	3.5	Valid
**152**	$\left\{MF_{18,}USC_{6}\right\}$	$\to \left\{N-\left\{MF_{18,}USC_{6}\right\}\right\}$	28.57%	100%	3.5	Valid
**153**	$\left\{MF_{18,}USC_{9}\right\}$	$\to \left\{N-\left\{MF_{18,}USC_{9}\right\}\right\}$	28.57%	100%	3.5	Valid
**154**	$\left\{MF_{18,}USC_{23}\right\}$	$\to \left\{N-\left\{MF_{18,}USC_{23}\right\}\right\}$	28.57%	100%	3.5	Valid
**155**	$\left\{MF_{18,}USC_{32}\right\}$	$\to \left\{N-\left\{MF_{18,}USC_{32}\right\}\right\}$	28.57%	100%	3.5	Valid
**156**	$\left\{MF_{18,}WUA2\right\}$	$\to \left\{N-\left\{MF_{18,}WUA2\right\}\right\}$	28.57%	100%	3.5	Valid
**157**	$\left\{MF_{18,}WUA3\right\}$	$\to \left\{N-\left\{MF_{18,}WUA3\right\}\right\}$	28.57%	100%	3.5	Valid
**158**	$\left\{MF_{18,}WUA10\right\}$	$\to \left\{N-\left\{MF_{18,}WUA10\right\}\right\}$	28.57%	100%	3.5	Valid
**159**	$\left\{MF_{18,}WUA11\right\}$	$\to \left\{N-\left\{MF_{18,}WUA11\right\}\right\}$	28.57%	100%	3.5	Valid
**160**	$\left\{MF_{18,}WUA12\right\}$	$\to \left\{N-\left\{MF_{18,}WUA12\right\}\right\}$	28.57%	100%	3.5	Valid
**161**	$\left\{MF_{18,}WUA19\right\}$	$\to \left\{N-\left\{MF_{18,}WUA19\right\}\right\}$	28.57%	100%	3.5	Valid
**162**	$\left\{MF_{18,}WUA20\right\}$	$\to \left\{N-\left\{MF_{18,}WUA20\right\}\right\}$	28.57%	100%	3.5	Valid
**163**	$\left\{MF2_{1,}USC_{1}\right\}$	$\to \left\{N-\left\{MF2_{1,}USC_{1}\right\}\right\}$	28.57%	66.67%	2.3	Valid
**165**	$\left\{MF2_{1,}USC_{6}\right\}$	$\to \left\{N-\left\{MF2_{1,}USC_{6}\right\}\right\}$	28.57%	100%	3.5	Valid
**169**	$\left\{MF2_{1,}WUA2\right\}$	$\to \left\{N-\left\{MF2_{1,}WUA2\right\}\right\}$	28.57%	100%	3.5	Valid
**173**	$\left\{MF2_{1,}WUA12\right\}$	$\to \left\{N-\left\{MF2_{1,}WUA12\right\}\right\}$	28.57%	100%	3.5	Valid
**176**	$\left\{USC_{1,}USC3\right\}$	$\to \left\{N-\left\{USC_{1,}USC3\right\}\right\}$	28.57%	66.67%	2.3	Valid
**177**	$\left\{USC_{1,}USC6\right\}$	$\to \left\{N-\left\{USC_{1,}USC6\right\}\right\}$	28.57%	100%	3.5	Valid
**178**	$\left\{USC_{1,}USC9\right\}$	$\to \left\{N-\left\{USC_{1,}USC9\right\}\right\}$	28.57%	66.67%	2.3	Valid
**179**	$\left\{USC_{1,}USC23\right\}$	$\to \left\{N-\left\{USC_{1,}USC23\right\}\right\}$	28.57%	66.67%	2.3	Valid
**180**	$\left\{USC_{1,}USC32\right\}$	$\to \left\{N-\left\{USC_{1,}USC32\right\}\right\}$	28.57%	66.67%	2.3	Valid
**181**	$\left\{USC_{1,}WUA2\right\}$	$\to \left\{N-\left\{USC_{1,}WUA2\right\}\right\}$	28.57%	66.67%	2.3	Valid
**182**	$\left\{USC_{1,}WUA3\right\}$	$\to \left\{N-\left\{USC_{1,}WUA3\right\}\right\}$	28.57%	100%	3.5	Valid
**183**	$\left\{USC_{1,}WUA10\right\}$	$\to \left\{N-\left\{USC_{1,}WUA10\right\}\right\}$	28.57%	66.67%	2.3	Valid
**184**	$\left\{USC_{1,}WUA11\right\}$	$\to \left\{N-\left\{USC_{1,}WUA11\right\}\right\}$	28.57%	66.67%	2.3	Valid
**185**	$\left\{USC_{1,}WUA12\right\}$	$\to \left\{N-\left\{USC_{1,}WUA12\right\}\right\}$	28.57%	100%	3.5	Valid
**186**	$\left\{USC_{1,}WUA19\right\}$	$\to \left\{N-\left\{USC_{1,}WUA19\right\}\right\}$	28.57%	66.67%	2.3	Valid
**187**	$\left\{USC_{1,}WUA20\right\}$	$\to \left\{N-\left\{USC_{1,}WUA20\right\}\right\}$	28.57%	66.67%	2.3	Valid
**188**	{USC3,USC6}	→{N−{USC3,USC6}}	28.57%	100%	3.5	Valid
**193**	{USC3,WUA3}	→{N−{USC3,WUA3}}	28.57%	100%	3.5	Valid
**196**	{USC3,WUA12}	→{N−{USC3,WUA12}}	28.57%	100%	3.5	Valid
**199**	{USC6,USC9}	→{N−{USC6,USC9}}	28.57%	100%	3.5	Valid
**200**	{USC6,USC23}	→{N−{USC6,USC23}}	28.57%	100%	3.5	Valid
**201**	{USC6,USC32}	→{N−{USC6,USC32}}	28.57%	100%	3.5	Valid
**202**	{USC6,WUA2}	→{N−{USC6,WUA2}}	28.57%	100%	3.5	Valid
**203**	{USC6,WUA3}	→{N−{USC6,WUA3}}	28.57%	100%	3.5	Valid
**204**	{USC6,WUA10}	→{N−{USC6,WUA10}}	28.57%	100%	3.5	Valid
**205**	{USC6,WUA11}	→{N−{USC6,WUA11}}	28.57%	100%	3.5	Valid
**206**	{USC6,WUA12}	→{N−{USC6,WUA12}}	28.57%	100%	3.5	Valid
**207**	{USC6,WUA19}	→{N−{USC6,WUA19}}	28.57%	100%	3.5	Valid
**208**	{USC6,WUA20}	→{N−{USC6,WUA20}}	28.57%	100%	3.5	Valid
**212**	{USC9,WUA3}	→{N−{USC9,WUA3}}	28.57%	100%	3.5	Valid
**215**	{USC9,WUA12}	→{N−{USC9,WUA12}}	28.57%	100%	3.5	Valid
**220**	{USC23,WUA3}	→{N−{USC23,WUA3}}	28.57%	100%	3.5	Valid
**223**	{USC23,WUA12}	→{N−{USC23,WUA12}}	28.57%	100%	3.5	Valid
**227**	{USC32,WUA3}	→{N−{USC32,WUA3}}	28.57%	100%	3.5	Valid
**230**	{USC32,WUA12}	→{N−{USC32,WUA12}}	28.57%	100%	3.5	Valid
**233**	{WUA2,WUA3}	→{N−{WUA2,WUA3}}	28.57%	100%	3.5	Valid
**236**	{WUA2,WUA12}	→{N−{WUA2,WUA12}}	28.57%	100%	3.5	Valid
**239**	{WUA3,WUA10}	→{N−{WUA3,WUA10}}	28.57%	100%	3.5	Valid
**240**	{WUA3,WUA11}	→{N−{WUA3,WUA11}}	28.57%	100%	3.5	Valid
**241**	{WUA3,WUA12}	→{N−{WUA3,WUA12}}	28.57%	100%	3.5	Valid
**242**	{WUA3,WUA19}	→{N−{WUA3,WUA19}}	28.57%	100%	3.5	Valid
**243**	{WUA3,WUA20}	→{N−{WUA3,WUA20}}	28.57%	100%	3.5	Valid
**245**	{WUA10,WUA12}	→{N−{WUA10,WUA12}}	28.57%	100%	3.5	Valid
**248**	{WUA11,WUA12}	→{N−{WUA11,WUA12}}	28.57%	100%	3.5	Valid
**251**	{WUA12,WUA19}	→{N−{WUA12,WUA19}}	28.57%	100%	3.5	Valid
**252**	{WUA12,WUA20}	→{N−{WUA12,WUA20}}	28.57%	100%	3.5	Valid

Rules 29, 33, 38, and 41 indicate that 100% of fatal construction accidents may be attributable to a combination of employment of unskilled personnel (MF_1_), deployment of faulty/unsafe tools, vehicles, and machines (MF_18_), faulty hoisting or lifting equipment or machine (USC_6_), non-compliance with hoisting or lifting procedures (WUA_3_) and operating equipment or machines without qualification or authorization (WUA_12_). Rule 31 indicates that 66.67% of fatal construction accidents are due to the employment of unskilled personnel (MF_1_) and a collapse in scaffolding/working platforms (USC_1_). Rules 49, 53, 58, and 61 indicate that 100% of fatal construction accidents could be attributable to a combination of not identifying, assessing, and controlling risks (MF_2_), deployment of faulty/unsafe tools, vehicles, and machines (MF_18_), faulty hoisting or lifting equipment or machine (USC_6_), non-compliance with hoisting or lifting procedures (WUA_3_) and operating equipment or machines without qualification or authorization (WUA_12_). Rule 51 indicates that 66.67% of fatal construction accidents are due to not identifying, assessing, and controlling risks (MF_2_) and a collapse in scaffolding/working platforms (USC_1_). Rules 68, 72, 77, and 80 indicate that 100% of fatal construction accidents may be attributable to a combination of financial constraints (MF_5_), deployment of faulty/unsafe tools, vehicles, and machines (MF_18_), faulty hoisting or lifting equipment or machine (USC_6_), non-compliance with hoisting or lifting procedures (WUA_3_) and operating equipment or machines without qualification or authorization (WUA_12_). Rule 70 indicates that 66.67% of fatal construction accidents may be due to financial constraints (MF_5_) and scaffolding/working platforms collapse (USC_1_).

Rules 86, 90, 94, and 88 indicate that 100% of fatal construction accidents are attributable to a combination of lack of safety regulations and enforcement (MF_5_), deployment of faulty/unsafe tools, vehicles, and machines (MF_18_), faulty hoisting or lifting equipment or machine (USC_6_), non-compliance with hoisting or lifting procedures (WUA_3_) and Operating machines without permission (WUA_12_). Rule 88 indicates that 66.67% of fatal construction accidents are due to a lack of safety regulations and enforcement (MF_12_) and a collapse in scaffolding/working platforms (USC_1_). Rules 103, 107, 111, and 115 indicate that 100% of fatal construction accidents are attributable to a combination of lack of safety training (MF_13_ deployment of faulty/unsafe tools, vehicles, and machines (MF_18_), faulty hoisting or lifting equipment or machine (USC_6_), non-compliance with hoisting or lifting procedures (WUA_3_) and Operating machines without permission (WUA_12_). Rule 105 indicates that 66.67% of fatal construction accidents are due to a lack of safety training (MF_13_) and scaffolding/working platforms collapse (USC_1_). Rules 119, 123, 128, and 131 indicate that 100% of fatal construction accidents could be attributable to a combination of lack of strict on-site safety supervision and poor management (MF_14_), deploying faulty/unsafe tools, vehicles, and machines (MF_18_), faulty hoisting or lifting equipment or machine (USC_6_), non-compliance with hoisting or lifting procedures (WUA_3_) and operating equipment or machines without qualification or authorization (WUA_12_). Rule 121 indicates that 66.67% of fatal construction accidents are due to a lack of strict on-site safety supervision and poor management (MF_14_), and scaffolding/working platforms collapse (USC_1_). Rules 134, 138, 143, and 146 indicate that 100% of fatal construction accidents are attributable to a combination of inadequate provision of safety equipment (MF_16_), deployment of faulty/unsafe tools, vehicles, and machines (MF_18_), faulty hoisting or lifting equipment or machine (USC_6_), non-compliance with hoisting or lifting procedures (WUA_3_) and Operating machines without permission (WUA_12_). Rule 136 indicates that 66.67% of fatal construction accidents could be because the required PPE for the job was not provided (MF_16_) and scaffolding/working platforms collapsed (USC_1_).

Rules 149–162 indicate that 100% of fatal construction accidents may be because of a combination of MF_18_, dangerous working, and lack of stringent operating procedures (MF_21_), scaffolding/working platforms collapsed (USC_1_), workplace congestedness (USC_3_), hoisting or lifting equipment or machine failure (USC_6_), defective tools/equipment/supplies/personal protective equipment (USC_9_), poor housekeeping (USC_23_), and unsafe working and operating procedures (USC_32_). Other factors include carelessness/negligence (WUA_2_), non-compliance with hoisting or lifting procedures (WUA_3_), personal qualities (such as a worker's safety attitude) (WUA_10_), insufficient safety understanding and misjudging dangerous scenarios (WUA_11_), operating machines without permission (WUA_12_), incorrect or inadequate personal protective equipment use (WUA_19_) and utilization of risky methods or procedures (WUA_20_). Rule 163 indicates that 66.67% of fatal construction accidents may be due to unsafe working conditions, a lack of stringent operating procedures (MF_21_), and scaffolding/working platforms collapse (USC_1_). Rules 165, 169, and 173 indicate that 100% of fatal construction accidents are due to a combination of unsafe working and lack of stringent operating procedures (MF_21_), faulty hoisting or lifting equipment or machine (USC_6_), carelessness/negligence (WUA_2_), and operating machines without permission (WUA_12_).

Rules 176, 178, 179, 180, 181, 183, 184, 186, and 187 indicate that 66.67% of fatal construction accidents are due to a combination of scaffolding/working platforms collapse (USC_1_), workplace congestedness (USC_3_), faulty tools/equipment/supplies/PPE (USC_9_), poor housekeeping (USC_23_), unsafe working and operating procedures (USC_32_), carelessness/negligence (WUA_2_), personal qualities (such as a worker's safety attitude) (WUA_10_), insufficient safety understanding and misjudging dangerous scenarios (WUA_11_), incorrect or inadequate personal protective equipment use (WUA_19_) and utilization of risky methods or procedures (WUA_20_). Rules 177, 182, and 185 indicate that 100% of fatal construction accidents are due to a combination of scaffolding/working platforms collapse (USC_1_), hoisting or lifting equipment or machine failure (USC_6_), non-compliance with hoisting or lifting procedures (WUA_3_), and operating machines without permission (WUA_12_). Rules 188, 193, and 196 indicate that 100% of fatal construction accidents are due to a combination of workplace congestion (USC_3_), hoisting or lifting equipment or machine failure (USC_6_), non-compliance with hoisting or lifting procedures (WUA_3_), and operating machines without permission (WUA_12_).

Rules 199–208 indicate that 100% of fatal construction accidents are due to a combination of fixing machines or equipment while in motion (USC_6_), faulty tools/equipment/supplies/PPE (USC_9_), poor housekeeping (USC_23_), unsafe working, and operating procedures (USC_32_), carelessness/negligence (WUA_2_), non-compliance with hoisting or lifting procedures (WUA_3_), personal qualities (such as a worker's safety attitude) (WUA_10_), insufficient safety understanding and misjudging dangerous scenarios (WUA_11_), operating machines without permission (WUA_12_), incorrect or inadequate personal protective equipment use (WUA_19_) and utilization of risky methods or procedures (WUA_20_). Rules 212 and 215 indicate that 100% of fatal construction accidents are due to a combination of faulty tools/equipment/supplies/PPE (USC_9_), non-compliance with hoisting or lifting procedures (WUA_3_), and operating equipment or machines without qualification or authorization (WUA_12_). Rules 220 and 223 also indicate that 100% of fatal construction accidents are due to a combination of poor housekeeping (USC_23_), non-compliance with hoisting or lifting procedures (WUA_3_), and operating equipment or machines without qualification or authorization (WUA_12_). Rules 227 and 230 also indicate that 100% of fatal construction accidents are due to a combination of unsafe working and operating procedures (USC_32_), non-compliance with hoisting or lifting procedures (WUA_3_), and using equipment or machines without qualification or authorization (WUA_12_).

Rules 233 and 236 also indicate that 100% of fatal construction accidents are due to a combination of carelessness/negligence (WUA_2_) and non-compliance with hoisting or lifting procedures (WUA_3_), and operating equipment or machines without qualification or authorization (WUA_12_).

Rules 239–243 indicate that 100% of fatal construction accidents are due to a combination of non-compliance with hoisting or lifting procedures (WUA_3_), personal qualities (such as a worker's safety attitude) (WUA_10_), insufficient safety understanding and misjudging dangerous scenarios (WUA_11_), operating machines without permission (WUA_12_), incorrect or inadequate personal protective equipment use (WUA_19_) and use of hazardous methods or procedure (WUA_20_). Rule 245 indicates that 100% of fatal construction accidents are due to personal qualities (such as a worker's safety attitude) (WUA_10_) and operating machines without permission (WUA_12_). Rule 248 indicates that 100% of fatal construction accidents are due to a lack of safety knowledge and misjudgment of potentially hazardous situations (WUA_11_) and operating machines without permission (WUA_12_). Rules 251 and 252 indicate that 100% of fatal construction accidents may be attributable to operating equipment or machines without qualification or permission (WUA_12_), incorrect or inadequate personal protective equipment use (WUA_19_) and utilization of risky methods or procedures (WUA_20_). The authors argue that the generated rules could be reliable, considering where the data used in this study was obtained.

➢ For every nonempty subset ‘N’ of ‘K’, the association rule is
➢ N → (K–N), If support (K)/support (N) ≥ min_confidence
➢ Minimum support = 2 (28.57%), minimum confidence = 60%

### 4.2 Discussion and implications of the results obtained

The mined association rules are concise summaries of the dataset's implicit rules. Eighty-two of the rules generated are 100% valid, and 18 are 66.67% correct. And the lift of those 82 rules is 3.5, and that of the 18 is 2.3. Identifying the primary factors contributing to construction accidents is the first step in minimizing and reducing accidents and their repercussions. This article's analysis demonstrates that management factors, hazardous site conditions, and unsafe worker behavior all influence the occurrence of fatal construction accidents. Most occupational injuries and deaths are avoidable because they are caused by the dangerous acts of the employees, management failures [10], and unsafe acts by workers [88]. This is in line with the primary findings of the research.

In contrast to this study's findings, some researchers found that weather conditions and seasonal variation significantly affect the safety performance of construction sites by altering the work environment and the requirements of the workers [9]. This could be because of the difference in weather conditions. The study has also identified the specific causes and patterns of deadly accidents in the Malaysian construction industry. MF_1_, MF_2_, MF_5_, MF_12_, MF_13_, MF_14_, MF_16_, MF_18_, and MF_21_ are the typical management factors that could influence the occurrence of fatal construction accidents. Workers' dangerous actions are errors that surpass a certain threshold of tolerance, misjudgment of hazardous situations, and intentional violations. The actions depend on the tasks carried out by the construction worker(s) and individual characteristics. WUA_2_, WUA_3_, WUA_10_, WUA_11_, WUA1_2_, WUA_19_, and WUA_20_ are the typical workers' unsafe actions that could influence the occurrence of fatal construction accidents. USC_1_, USC_3_, USC_6_, USC_9_, USC_23_, and USC_32_ are the customary dangerous site conditions that could impact the event of fatal construction accidents based on the generated valid rules.

As a result, attention should be given to the three identified fundamental factors that trigger various construction accidents to mitigate their occurrence on-site. In these circumstances, the primary cause-and-effect relationships are between the sub-factors (i.e., management factors, unsafe site conditions, and workers' unsafe actions).

## 5 Conclusion

The immediate requirement to transform the causes of accidents into valuable information and knowledge may be addressed by data mining. Association rule mining was employed in this research using an apriori algorithm to determine the affinity between the sub-factors that cause fatal accidents in the Malaysian construction industry. Two hundred and fifty-three (253) rules were generated, and a hundred (100) demonstrate strong relationships among the sub-factors. Association rule mining can help researchers better understand and manage accidents [4]. However, readers should be aware that, while association rule mining can identify previously unknown associations, it can also create many obvious or meaningless rules [9,10]. Our analysis uncovered several unexpected, latent, and previously undiscovered traits and patterns associated with the specific causes of fatal construction accidents based on three fundamental causes. The study concludes that fatal construction accidents are caused by management factors, hazardous site conditions, and workers' risky behaviours, and that the level of safety in the construction industry is strongly dependent on these three crucial aspects [88].

The results can be used to identify the hidden patterns or knowledge, assisting in building a safer working environment in the construction sector. The study findings can be used to design effective inspection procedures and occupational safety initiatives more efficiently. Additionally, the study's findings are expected to serve as a foundation for decision-making on accident prevention and control in Malaysia's construction industry and other countries with similar characteristics. This research also made it possible to better understand the relationship between construction accident causes and lay the theoretical groundwork for further investigation. Finally, the proposed method should be tested in a broader range of construction situations and scenarios to ensure that it is as accurate as possible. This study's findings are based on fatal construction data obtained from the DOSH website. The only data analyzed is from Malaysia, so the conclusions may not apply to other nations, but they can provide indications of different criteria that are standard. Such studies could yield more knowledge if richer data for accidents were collected.

## Author contribution statement

Aminu Darda'u Rafindadi, Nasir Shafiq, Idris Othman, Ahmad Ibrahim, M. M. Aliyu, Miljan Mikić and Hamzh Alarifi: Conceived and designed the experiments; Performed the experiments; Analyzed and interpreted the data; Contributed reagents, materials, analysis tools or data; Wrote the paper.

## Data availability statement

The datasets are available from the corresponding author on reasonable request.

## Funding statement

This research did not receive any specific grant from funding agencies in the public, commercial, or not-for-profit sectors.

## Additional information

No additional information is available for this paper.

## Declaration of competing interest

The authors declare no conflict of interest.

## Appendix A Supplementary data

Addition and retention of authorship Addition and retention of authorship
