# Supplementary material for: Data mining of the essential causes of different types of fatal construction accidents
Source: Heliyon. 2023 Feb 2;9(2):e13389. doi: 10.1016/j.heliyon.2023.e13389 (PMC9905945; doi:10.1016/j.heliyon.2023.e13389)
Supplement: Addition and retention of authorship [file mmc1.pdf]

24/11/2022

Dear Editor,

Addition and retention of authorship

The authors have agreed to add Dr M. M. Aliyu and retain Dr Miljan Mikić to the authorship list. Also, all authors agree to the author order as follows: Aminu Darda'u Rafindadi, Nasir Shafiq, Idris Othman, Ahmad Ibrahim, M. M. Aliyu, Miljan Mikić, Hamzh Alarifi. Please find below the authors’ signatures with dates.

S/No	Name of authors	Statement	Signature	Date
1	Aminu Darda’u Rafindadi	I agree with the proposed new authorship addition of M.M. Aliyu and retain Dr Miljan Mikić to the list.	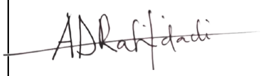	24/11/2022
2	Nasir Shafiq	I agree with the proposed new authorship addition of M.M. Aliyu and retain Dr Miljan Mikić to the list.	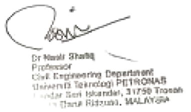	24/11/2022
3	Idris Othman	I agree with the proposed new authorship addition of M.M. Aliyu and retain Dr Miljan Mikić to the list.	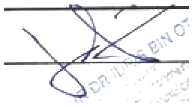	25/11/2022
4	Ahmad Ibrahim	I agree with the proposed new authorship addition of M.M. Aliyu and retain Dr Miljan Mikić to the list.	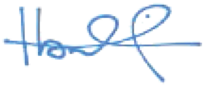	24/11/2022
5	M. M. Aliyu	I agree with the proposed new authorship addition of my name and retention of Dr Miljan Mikić to the list.	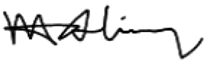	24/11/2022
6	Miljan Mikić	I agree with the proposed new authorship addition of M.M. Aliyu and retain Dr Miljan Mikić to the list.	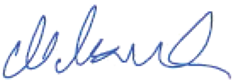	25/11/2022
7	Hamzh Alarifi	I agree with the proposed new authorship addition of M.M. Aliyu and retain Dr Miljan Mikić to the list.	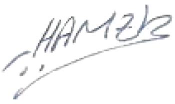	25/11/2022

Sincerely,

Aminu, on behalf of other authors
